# ‘It All Kind of Links Really’: Young People’s Perspectives on the Relationship between Socioeconomic Circumstances and Health

**DOI:** 10.3390/ijerph19063679

**Published:** 2022-03-19

**Authors:** Hannah Fairbrother, Nicholas Woodrow, Mary Crowder, Eleanor Holding, Naomi Griffin, Vanessa Er, Caroline Dodd-Reynolds, Matt Egan, Karen Lock, Steph Scott, Carolyn Summerbell, Rachael McKeown, Emma Rigby, Phillippa Kyle, Elizabeth Goyder

**Affiliations:** 1 Health Sciences School, University of Sheffield, Sheffield S10 2LA, UK; 2 ScHARR, University of Sheffield, Sheffield S1 4DA, UK; n.woodrow@sheffield.ac.uk (N.W.); m.crowder@sheffield.ac.uk (M.C.); e.holding@sheffield.ac.uk (E.H.); e.goyder@sheffield.ac.uk (E.G.); 3 Department of Sport and Exercise Science, Fuse|Durham University, Durham DH1 3HN, UK; naomi.c.griffin@durham.ac.uk (N.G.); caroline.dodd-reynolds@durham.ac.uk (C.D.-R.); carolyn.summerbell@durham.ac.uk (C.S.); phillippa.kyle@durham.ac.uk (P.K.); 4 Department of Health Services Research and Policy, London School of Hygiene and Tropical Medicine, London WC1E 7HT, UK; vanessa.er@lshtm.ac.uk (V.E.); matt.egan@lshtm.ac.uk (M.E.); karen.lock@lshtm.ac.uk (K.L.); 5 Population Health Sciences Institute, Faculty of Medical Sciences, Newcastle University, Newcastle upon Tyne NE1 4LP, UK; steph.scott@newcastle.ac.uk; 6 Association for Young People’s Health, London SE1 4YR, UK; rachael@youngpeopleshealth.org.uk (R.M.); emma@youngpeopleshealth.org.uk (E.R.)

**Keywords:** health inequalities, social inequalities, social determinants of health, young people, qualitative

## Abstract

Meaningful inclusion of young people’s perceptions and experiences of inequalities is argued to be critical in the development of pro-equity policies. Our study explored young people’s perceptions of what influences their opportunities to be healthy within their local area and their understandings of health inequalities. Three interlinked qualitative focus group discussions, each lasting 90 to 100 min, with the same six groups of young people (*n* = 42) aged 13–21, were conducted between February and June 2021. Participants were recruited from six youth groups in areas of high deprivation across three geographical locations in England (South Yorkshire, the North East and London). Our study demonstrates that young people understand that health inequalities are generated by social determinants of health, which in turn influence behaviours. They highlight a complex interweaving of pathways between social determinants and health outcomes. However, they do not tend to think in terms of the social determinants and their distribution as resulting from the power and influence of those who create and benefit from health and social inequalities. An informed understanding of the causes of health inequalities, influenced by their own unique generational experiences, is important to help young people contribute to the development of pro-equity policies of the future.

## 1. Introduction

There is a well-established relationship between socioeconomic position and health [1,2]. Health follows a socioeconomic gradient, where each step up the socioeconomic ladder is associated with better outcomes [3,4]. This patterning is longstanding and evident throughout the life course across a range of different outcomes at both micro and macro geographical levels [5,6,7]. In the UK, set against a backdrop of rising levels of poverty and the fallout of government austerity policies following the 2008 recession [8], the past decade has seen socioeconomically patterned health inequalities widen for both adults and children and young people [9,10]. More recently, the COVID-19 pandemic has exacerbated existing inequalities, with those lower down the socioeconomic ladder being disproportionately affected both in economic and health terms [11]. 

Central to many contemporary explanations for socioeconomically patterned health inequalities is the concept of Social Determinants of Health (SDH). The World Health Organisation’s Commission on the Social Determinants of Health described the SDH as ‘the conditions in which people are born, grow, live, work and age’ and argued that ‘the marked health inequities between countries are caused by the unequal distribution of power, income, goods, and services, globally and nationally’ [7] (p. 1). However, while there is broad consensus as to the importance of the SDH, there is much less consistency in the way in which the concept is mobilised [12,13,14]. What we see is a range of discourses that draw upon the SDH but differ significantly in the way they explain *how* societal factors result in differences in health [15]. These differences in interpretation, Raphael (2011) argues, are not just about ‘intellectual world views’ but fundamentally affect how we seek to approach and redress health inequalities [12] (p. 223). Raphael (2011) proposes a spectrum of seven discourses to encapsulate the different ways of understanding (and responding to) the SDH (see Table 1) [12]. 

According to Raphael (2011) and other key researchers in the field (such as Scott-Samuel and Smith (2015), a real reduction in apparently intractable health inequalities will only be possible by tackling inequitable political structures and the power and influence of the people that shape them (Discourse Level Six and Seven) [12,16]. To create a step-change, Raphael argues, we need to ‘educate […] the public that deteriorating quality SDH and inequitable SDH distributions result from the undue influence upon public policymaking of those creating and profiting from social and health inequalities’ [12] (p. 230). The argument that we need to change public understandings is widespread [17] and reinforced by a recent collaboration between the Health Foundation and the Frameworks Institute, which sought to ‘develop a deeper appreciation of the ways in which people understand and think about health in order to develop more effective approaches to communicating the evidence’ [18]. Improving public awareness of health inequalities and the social determinants of health is argued to be vital for galvanizing support for change to the political status quo and the development of pro-equity policies [18]. 

Studies exploring public perceptions of the link between socioeconomic circumstance and health, however, are limited [17,19]. There is broad agreement regarding the importance of the SDH among the research community, with many narratives echoing the higher-level discourse of Raphael’s (2011) typology through repeated critiques of a focus on lifestyle behaviours and neglect of the causal pathways of health inequalities and economic and environmental factors [12,20,21]; though see Dijkstra and Horstman’s (2021) critique of social epidemiological research which constructs low socioeconomic status populations as ‘inherently unhealthy and problematic’ [22] (p. 6). However, public understanding of the factors shaping health has been argued to be limited [12,17,23]. This is supported by recent research by the Frameworks Institute which found that ‘public discourse and policy action is limited in acknowledging the role that societal factors such as housing, education, welfare and work play in shaping people’s long-term health’ [18] (p. 1). Drawing on the Frameworks Institute’s findings on young people’s views, Marmot et al. (2020) characterised public understandings as individualistic, fatalistic and prone to divisive ‘them and us’ thinking [1] (p. 145). In contrast, in their review of the admittedly limited evidence base (a meta-ethnography of 17 qualitative studies), Smith and Anderson (2018) argue that people experiencing socioeconomic disadvantage do display an awareness of how socioeconomic hardship can lead to ill health [17]. The picture is thus mixed with contradictory findings regarding the perceived chasm between research consensus and public understanding. In the context of increasing socioeconomic and health inequalities over recent decades and particularly recently due to the COVID-19 pandemic (which has exacerbated existing, socially patterned inequalities through its interaction with inequalities in chronic disease and the social determinants of health including poor quality housing and lower access to healthcare in disadvantaged communities) [1,11,24], it is an opportune time to revisit public perceptions of how socioeconomic circumstances shape health. Further, Smith and Anderson (2018) highlight a dearth of studies exploring the views and experiences of young people [17] (see also Woodgate and Leach’s (2010) study and Backett-Milburn et al.’s 2003 study [25,26]). This is an important gap in the evidence base [17,22,27,28]. Youth activism in other spheres such as climate change teaches us that young people have the potential to galvanize support for and contribute to significant policy change [29]. 

### Study Aim

The aim of our research project was to explore young people’s perceptions of what influences their opportunities to be healthy within their local area and their understandings of health inequalities. This paper presents key findings on young people’s perspectives on the relationship between socioeconomic circumstances and health.

## 2. Materials and Methods

### 2.1. Overview 

We undertook a series of three interlinked qualitative focus group discussions with six groups of young people (*n* = 42) aged 13–21, resulting in 18 focus group discussions in total. Participants were recruited from six youth groups across three geographical locations in England (South Yorkshire (SY), the North East (NE) and London (L)). All three locations fell within the most deprived quintile based on the 2019 English indices of multiple deprivation (IMD). Data generation took place between February and June 2021, during the COVID-19 pandemic. Due to the UK’s lockdown and social distancing restrictions [30], the majority of focus groups were conducted online (*n* = 15). However, focus group discussions (*n* = 3) with one youth group in the North East were conducted face-to-face, once social distancing restrictions permitted, since the youth group did not have facilities to support online data generation in their building (e.g., computers, Wi-Fi) and not all the young people had the technologies to participate from home. Focus group discussions lasted between 90 and 100 min. Further details on the methodological and ethical challenges of this study are described elsewhere [31]. Ethical approval for the study was granted by the School of Health and Related Research (ScHARR) Ethics Committee at the University of Sheffield. 

Throughout our project, we actively engaged with Mason’s (2018) ‘difficult questions’ for qualitative research to help ensure the quality, rigour and methodological integrity of our study [32]. In relation to reliability, we seek to provide a detailed and transparent account of our sampling, recruitment, data generation and analysis. Our concerns for the validity of our method and interpretation focus on ensuring the fit between our method and ‘tracing the route’ (albeit a messy and non-linear one) of our interpretations. 

### 2.2. Sampling and Recruitment 

The focus groups involved young people from pre-existing youth organisations, and our sampling was shaped by each group’s demographics. Given our focus on socioeconomic circumstances, we initially sought to work with youth groups in socioeconomically contrasting areas. Recognising that socioeconomic position permeates and intersects with other axes of inequality, we also sought to ensure that we worked with young people of different genders and ethnicities in both urban and rural areas (including coastal areas), and we approached youth groups that we thought would enable this. However, due to challenges of recruitment during a pandemic (with youth groups pausing and/or moving online) we had to take a pragmatic approach and work with youth groups with whom we already had established working relationships, all of which were in areas of high deprivation. The youth group workers we approached saw issues around health inequalities as pertinent in their areas and thus important to engage with. Further, while we initially aimed to work with young people aged 13–17, we took a flexible, inclusive approach as some of our youth groups also included young people over 18. We did not want to exclude young people outside of this range if they were keen to participate, particularly since the focus groups replaced their usual weekly meetings. Our inclusive approach also recognises that young people’s transitions to adulthood in the UK have become increasingly elongated and less linear [33]. It is important to understand the concepts of ‘youth’ and ‘adulthood’ as not being simply a feature of age but also encompassing a variety of different experiences and understandings within this life phase [34]. The young people we worked with were all members of youth organisations, which, for us, was a primary criterion for participating in this study. 

In this way, we adopted a purposive sampling strategy, designed to encapsulate a relevant range of perspectives [32]. Drawing on Braun and Clarke (2021), our sample was guided by the breadth and focus of the research question(s); the demands placed on participants; the depth of data likely to be generated; pragmatic constraints; and the analytic goals and purpose of the overall project [35]. Our approach coheres with Braun and Clarke’s (2021) description of qualitative research as a ‘situated, reflexive and theoretically embedded practice of knowledge generation’ [35] (p. 210). This focus on the active construction of meaning opens up the potential to keep working towards new understandings. Our final sample consisted of 42 young people aged 13–21 and included young people of different genders and ethnicities in both urban and rural areas (including coastal areas) (see Table 2). 

Youth workers invited group members to participate and shared an information video and project overview, and researchers attended sessions with the youth groups to talk through the study and build rapport. Any young person interested in taking part was given a more detailed information sheet. For potential participants under the age of 16, opt-in consent from parents/guardians was gained. Written consent was then gathered for all participating young people (either on paper or electronic). Participants were asked to provide basic demographic information, including their postcode, which we used to capture an overall average deprivation rank measure (average position out of the 32,844 small areas in England, with closer to 1 being more deprived) (see Table 2). Despite all the field sites falling in the most deprived quintile, the average participant position across the groups ranged from quintile 1 to 3.

### 2.3. Data Generation 

The stigmas around topics of health and inequality (where practices and situations are individualised and equated with deficit, passivity and irrational choice) make discussion of such topics challenging [36,37]. We employed focus group discussions to generate data and we gave careful consideration to the topic guides (activities and language used), as well as how support could be provided during and after the sessions [31]. While focus groups may prevent people sharing information due to concerns around privacy and stigma [38], they can help to reduce potential power differentials between researchers and participants and provide a space where people can discuss challenging topics with the support of others [39]. We ensured that we framed our questions so that participants could talk generally about young people in their area rather than feeling pressured into discussing their own personal experiences (e.g., ‘What kind of things where you live support young people to be ‘healthy’?’). Youth workers helped to facilitate the discussions alongside the research team. As well as having at least one youth worker involved in each session, we had four members of the research team in each online session and two members of the research team in each face-to-face session. There was at least one week between each of the three sessions for each group, which helped to avoid fatigue and to provide the opportunity for participants to reflect on and discuss the sessions with youth workers and peers.

Topic guides were piloted and revised as part of our Public Involvement and Engagement work with partner youth organisations (see Supplementary File S1: Topic Guides). Both online and face-to-face focus groups followed the same format (introductions, warm-up activity, main activity (in smaller breakout groups) and close and cool-down activity). The first focus group used a participatory concept mapping activity (for example see Jessiman et al. 2021 [40]) to explore perceptions of what influences young people’s opportunities to be healthy in their local area (see Supplementary File S2 for an example of a map developed from participants’ discussions). The second looked at understandings of inequalities in health. Participants were asked to discuss what they understood by the term ‘health inequality’ and asked to select and share their ideas about contemporary news articles relevant to health inequalities (e.g., free school meals, the uneven impact of COVID-19). The third focus group involved a discussion of the young people’s key priorities for change in improving health in their area. 

### 2.4. Data Analysis 

In keeping with our open question framing (designed to avoid participants feeling pressured into disclosing personal stories), we employed thematic analysis, drawing on Braun and Clarke’s (2006) framework [41]. In particular, our approach was guided by an emphasis on analysis as ‘creative and active’ [42] (p. 343) and an inherently ‘interpretive, reflexive process’ [42] (p. 332). The qualitative data management software system NVivo-12 was used to support data management. A coding frame (see Supplementary File S3: Coding Framework) was developed through HF, MC, VE, NG, EH and NW independently reading and adding descriptors to a selection of transcripts. Key codes and overarching themes were then discussed and agreed upon. The development of the coding framework was largely inductive, but an initial scaffolding was provided by key concepts in the literature and the research questions [17,43,44,45,46]. Following independent double coding of a selection of the transcripts (*n* = 6, two from each geographical area), we refined the framework before finally coding all transcripts. The development of a coding framework enabled multiple researchers in different locations to contribute to coding. The framework was used as a flexible starting point for analysis for this paper, which was carried out by the two lead authors (H.F. and N.W.). While we did not originally code our transcripts in relation to Raphael’s (2011) SDH discourse framework [12], we mobilise this framework in the discussion of our findings as a helpful tool for illuminating *how* young people understand the relationship between socioeconomic circumstances and health. 

We use verbatim extracts from the focus groups to illustrate the key findings. While we collected demographic data, this was anonymised at the point of collection to protect participant confidentiality. This means only the field site location and focus group session for each quote are provided (e.g., SY1.1 = South Yorkshire Group 1, session 1). Thus we are unable to identify individual quotes from participants but have endeavoured to present a range of young people’s voices across and within all participant groups.

## 3. Results

### 3.1. Perceptions of Factors Linking Socioeconomic Position and Health 

Participants in our study identified a number of different factors that they perceived to impact upon young people’s opportunities to enjoy good health in their local area. Through the course of their discussions, they described how abilities to eat healthily, access health-promoting spaces and activities and housing conditions all influenced health and were all shaped by socioeconomic position. The exacerbating impact of COVID-19 upon these abilities was also discussed. These themes were salient across all youth groups. 

#### 3.1.1. Eating Healthily in Contexts of Deprivation

Young people described a range of barriers to eating healthily in contexts of deprivation: the cost of and access to ‘healthy’ food, the apparent ubiquity of ‘unhealthy’ food, time pressure and competing priorities for limited financial resources. There was a general consensus among participants that ‘healthy food’ (particularly fresh fruit and vegetables) was more expensive than ‘unhealthy food’ (particularly processed foods) and that this was a key source of inequality:

*‘you can get chocolate bars for £1, you can get KFC for £1, £2 for a whole meal […] and then you go for the healthy meals and it’s like £3, £4 for no reason. And then they ask, oh why is everyone not eating healthy food instead? How can we eat healthy food if the area doesn’t even have any healthy food, it’s, our environment is just full of unhealthy food.’*

(L1.1)

Many young people described poor access to healthy food within their local areas and contrasted this with the ubiquity of ‘fast-food’ take-away outlets. Young people from London in particular linked fast-food density to the socioeconomic context of the area:

*‘when I go to richer parts of London, for example, like, when I go to the City where my university is, the [Name of university], I don’t see that many fast food shops around me but I see, like, when I’m in my own local area, [Name of location], there’s so many fast food shops.’*

(L2.2)

They also described how this situation had worsened due to the COVID-19 lockdown measures, with spaces seen as ‘unhealthy’ (takeaways) noted to be quicker to open up than health-supporting spaces (youth clubs, gyms). Generally, limited financial resources alongside limited access created a context of *unaffordability*, which constrained consumption choices. A number of participants challenged the notion that eating healthily is necessarily expensive and described how buying takeaways would be costly when looking at the perspective of buying every day and for a whole family. However, whilst some participants foregrounded the importance of behaviours such as cooking skills and planning meals ahead, others, through the group discussions, positioned individualised arguments like this against working families’ busy lives. They thought that people on a low income were also likely to be ‘time poor’ and that this would push them towards quicker and easier, but not healthier, ‘choices’: ‘*people on low incomes often, a lot of the time they work more hours and they can’t afford childcare and stuff so they don’t have the time to like prepare meals, which are like really healthy*’ (SY2.2). One participant eloquently explained how a lack of time ‘forced’ parents ‘*to do—in a way—irrational things, such as constantly sending a fast food order*’ (NE2.2). 

While in one group some young people initially found it difficult to understand higher rates of obesity among lower socioeconomic groups, through the course of their discussion, they made sense of the apparently counterintuitive link:

*Participant A You associate free school meals with poorer families who don’t necessarily have obesity, if you get me. So the people who have got the money buy the food and then eat it and then get obese. But for me it’s quite interesting that obviously obesity is associated with poorer families.*

*Participant B Healthier foods tend to be more expensive, like you can get one thing which is like full fat and it’ll be like £2 and if you want to get the fat-free version it’s like £3.50 or something.*

*Participant A Yeah, yeah, I was thinking the same as well. Obviously the cheaper stuff’s worse, if you get me, and more unhealthy.’*

(SY1.2)

Young people also highlighted competing priorities for people on a low income (e.g., household bills, activities, clothes), which meant that they could not always ‘choose’ the healthy option. A salient theme within many food narratives was the shame associated with the inability, or bounded ability, to ‘consume correctly’: ‘*When people have to buy cheaper options, sometimes they get ashamed quite a lot, people saying that they’re being right cheap or it’s bad things or they’re being lazy* […]’ (SY1.1). Indeed, some participants highlighted how food banks, designed to attenuate the impacts of poverty, could represent a source of embarrassment and shame for those who used them:

*‘there’s more food banks and stuff opening, which is a good thing, especially in this area but some people might be embarrassed to go to one because they don’t want to show that they’re in poverty…people might shame them for it, definitely…There’s like the ideal, you can provide for your family without any help or charity help, and people want to show to be like that and they don’t want people to see them as like not working and being lazy, which obviously is not going to be good on the mental health.’*

(SY2.1)

Such quotes highlight the importance young people attached to the shame associated with poverty, and the use of the word ‘lazy’ hints at their awareness of deficit discourses of the ‘undeserving poor’ [47]. In relation to food then, young people demonstrated nuanced understandings of the everyday challenges of life on a low income and described how different factors compounded each other. 

#### 3.1.2. Health-Promoting Spaces and Activities

Health-promoting spaces were generally described as places where young people could exercise and/or socialise, and participants related them to both physical and mental health. Opportunities to access and participate in health-promoting spaces and activities were often perceived to be strongly shaped by socioeconomic position. Many young people emphasised the high cost of access to activities and spaces (e.g., gyms, sports clubs), and participants in South Yorkshire and the North East also talked about the prohibitive cost of public transport to different leisure spaces (London participants highlighted that transport was free for young people). Personal accounts described how this played out: ‘*we did badminton for a while but then they made it £3 a night and barely anyone could afford it […] the whole club fell in on itself and stopped because people couldn’t pay to attend it*’ (SY1.1). Demonstrating a clear sense of injustice, one participant, talking about meeting at the local snooker hall, argued, ‘*some places can be unnecessarily expensive, and it’s not really fair on them though because like all we’s want to do is hang out with friends and we’s can’t get to it*’ (NE2.1). The move from ‘them’ to ‘we’ in the course of the short narrative serves to convey how this personally affects the participant and their friends and perhaps hints at how acutely young people experienced the unfairness here. Young people consistently contrasted expensive or inaccessible activities with their local youth groups which were perceived as providing a nearby safe, welcoming and affordable space to relax and socialise: ‘*[the youth group] is good for our health ‘cos we get to hang out with our friends and play out back*’ (NE2.1). Youth groups then were depicted as attenuating the impacts of poverty and socioeconomic disadvantage.

A small number of participants voiced a belief that, irrespective of income levels, young people could always use outdoor spaces for exercise. However, across all groups participants spoke of how perceptions of safety in their local areas were key inhibitors to accessing public spaces. Participants frequently described high levels of crime within their local areas compared to other places:

*‘So, when there are a lot of like stabbings going on in the area, people, like their parents won’t let them go outside […] so I think if crime could reduce in the area maybe people would have more access to these mental health spaces that are available.’*

(L1.1)

The phrase ‘these mental health spaces’ highlights young people’s emphasis on the potential for social spaces to positively impact upon their health and wellbeing. Parks were particularly singled out as places that young people could not enjoy to their full potential due to safety concerns: ‘*I live near a skate park and sometimes I get intimidated when I’m walking past because a lot of the time they’re doing like drugs, drinking. On a night time I wouldn’t want to be like round there*’ (SY2.2). Narratives about risk (crime and safety) were particularly common among female and LGBTQ+ participants across the different areas. While official supervision (e.g., security, police) was noted in some cases to help young people feel safer and support the use of such spaces, such supervision seemed to be rare. Indeed, for many participants, concerns about public anti-social behaviour, and especially the substance use of other people, was noted to shape perceptions and use of space. There was, however, an acknowledgement from some that ‘risky behaviours’ were also related to exclusion or a lack of activities for young people to engage in: ‘*if there’s nothing to do then we’re going to get ourselves into trouble*’ (SY1.1). The movement between describing ‘others’ in narratives about drug use in the local area and the ‘we’ and ‘ourselves’ in this extract affords a pertinent example of how participants moved between individualising, othering narratives to a more collective sense of the importance of socioeconomic circumstances in limiting opportunities.

#### 3.1.3. The Relationship between Poor Housing and Poor Health 

Young people highlighted the relationship between poor housing and poor health, particularly mental health:

*‘I feel like the housing is very cramped in the area, like it’s very cramped, like it’s very overcrowded and I feel like that also does have a big impact on mental health as well. Because it’s so overcrowded you don’t have any time to yourself, any time to think, literally with people around.’*

(L1.1)

Echoing the perceived shame associated with not eating ‘correctly’, participants described the shame related to living in the ‘wrong’ kind of housing: ‘*I’ve seen people who’ve felt embarrassed over it, not wanting to like invite friends over and then they’re just kind of feeling alone*’ (SY1.1). In this way, young people emphasised how poor housing had a significant impact on their social and emotional wellbeing—through everyday stress, embarrassment and reduced opportunities to socialise in one’s own home. Although much less salient in their narratives, they also discussed the relationship between housing and physical health. This was articulated particularly in relation to the COVID-19 lockdown measures, which meant people had to stay at home more than usual. Some described how ‘richer’ families could afford to purchase home exercise machines and contrasted this with poor people who had neither the financial resources nor space to do so: *‘So obviously some people might be in a small flat or whatever, no garden, they might not have the space to exercise either indoors or outdoors as such*’ (SY1.2).

### 3.2. Patterning and Pathways in Socioeconomic Inequalities in Health 

As well as highlighting specific factors linking socioeconomic position and health, young people voiced their understandings of how inequalities were patterned. They described both geographical (regional and localised) and intergenerational patterns of socioeconomic inequality. They also directly and indirectly emphasised the interrelationships between factors affecting health and the complexity of pathways between socioeconomic position and health outcomes. 

#### 3.2.1. Regional and Localised Inequalities

Regional inequalities were seen by the participants as underlying socioeconomic inequalities in health. Young people across all groups described a North–South divide in terms of wealth. The government was perceived to be responsible for creating and perpetuating this inequality through uneven investment, as articulated here by one of the London participants:

*‘I know that in north England [people] are not as wealthy as the south of England, kind of thing. Because obviously, like, the government, well, over the recent years the government’s basically just been focusing on the south of England because of, yeah, that’s where the capital is and it’s a bit more, the economy in the south of England’s a lot better than the north. So I guess, the pandemic has highlighted the fact that they’ve been, the government has, kind of, been putting the north on the side and just, like, yeah, not paying attention to their needs as much…I feel like as, like, as, like, as a whole that the south of England has just got more investment than the north of England.’*

(L2.2)

Young people vividly articulated how differences in local economies and labour markets between the North and South created tangible differences in everyday working and living conditions: ‘*Well there’s obviously more technical industries in London, so like engineering or ICT work. There isn’t those jobs in [South Yorkshire town]*’ (SY2.2). Local labour markets in the North were perceived to revolve around hospitality and service sector employment, which many associated with low pay, insecurity and low job satisfaction: ‘*the more like boring [jobs]*’ (SY2.1).

Focus group discussions often contained references to much more local-level inequalities too. In the following narrative, reference is made to a ‘clear split’ in wealth distribution between different areas:

*‘There’s certain parts of town where you can, they’re just known for people being either real poor there or they’re barely scraping by and there’s also bits where basically people who are wealthy live and it’s like quite a clear split. So all those people who live in the, I wouldn’t say dodgy areas but like with poorer people, they haven’t got as good quality of diet and stuff because they’re probably living off more cheaper meals that are just packed full of like chemicals or sugars and stuff.’*

(SY2.2)

The participant appears to show some awareness that people and places can be stigmatised and that lack of money and place-level disadvantage are barriers to healthy lifestyles. Hence the participant expresses unease about using the word ‘dodgy’ and appears to be avoiding individualistic, victim-blaming discourses. Some of the young people in London travelled to schools outside their local area, and this seemed to heighten their awareness of localised inequalities:

*‘The schools I have been to have generally been in wealthier areas than the area I live in and I’ve noticed that they definitely have a lot more green space and like generally just a lot more space within school to do sports and stuff as well, yeah…in like less like affluent places like there’s more like residential spaces and that’s, like people would say like [Name of location] has like an overcrowding like housing issue. And like I think like the main reason why is because in wealthier places like people are more spread out like the sort of like, on, like well people who are more affluent tend to have less children, people who are more affluent tend to like live out, more spaced out from each other… you can get stuck in a cycle because it’s so expensive in Central London so then because it’s so expensive you’re spending your money on other stuff you won’t be able to afford to move out to a wealthier area, where there’s like potentially, I don’t know more green space and less air pollution. So you can, yeah, you can just kind of get stuck in the, that cycle yeah.’*

(L2.1)

Here, however, the narrative moves from emphasizing environmental factors (access to green space and better housing) to behavioural factors (affluent families have fewer children) and then back to environmental factors (expensive housing, green space, air pollution). The narrative echoes the interplay and pull between different factors and exemplifies young people’s willingness to engage in complex understandings of causal pathways. Further, through their discussions, young people demonstrated an awareness of individualised discourses around blaming. They also consistently highlighted the injustice of the inequality that they perceived: ‘*It’s actually unfair. The facts are right there in front of your eyes, because if you’re born quite a poor person, then most people would expect you to stay poor and vulnerable to a lot of diseases*’ (NE2.2).

Many participants also discussed how substance use (tobacco smoking and drugs) was more prevalent in their area than other, more affluent areas. There was a suggestion that ‘other’ young people surrounded by drug taking and drinking would go on to engage in these behaviours themselves: ‘*Like round my area it’s quite bad for drugs and stuff like that…they see other people doing it, it’ll make them want to try it and then they’ll probably end up getting addicted and stuff like that*’ (SY1.1). However, the participants positioned themselves as avoiding the inevitability of this. Thus, they acknowledged structural issues and suggested deterministic outcomes for ‘other’ young people due to place-based disadvantage but discussed exercising their own agency to avoid this: ‘*Around my area it’s like the teens who are similar to my age have all gone mad with nights out and like drugs and that, so I won’t walk out. I see gangs and I’m like no, you’re not getting me*’ (SY1.1). 

#### 3.2.2. Intergenerational Patterns of Inequality

Young people repeatedly articulated the interactions between regional and intergenerational patterns of inequality and frequently commented on the presence and transfer of health-damaging practices through families and within communities. In the following narrative, one young person eloquently describes intergenerational continuity in practices and intergenerational cycles of poverty but also the inextricable link between the two:

*‘If they’re in a poor area, it’s much worse because their mum and dad might just give them a quid and tell them to go and buy their tea, instead of having like a home-cooked meal that’s full of good stuff. If that happens in one place, then it’ll start spreading in a way so more people will be getting poor because, like—let’s say one family, if they have two sons and those two sons have sons and they’re all like staying in the same area, it multiplies and then there’s like these areas where there’s shops and stuff and it’s all corner shops where they sell like ready meals and stuff and they all live off that and then they don’t have as good a diet, which isn’t their fault in the first place, it’s just where they were born and put into the world.’*

(SY2.2)

Highlighting the permeation of adverse health practices, there was an explanation of how health practices, such as diet, were shaped by experiences and exposure to parents’ and peers’ behaviours in a sociocultural context, which were described as ‘normalising’ such practices:

*‘Like we were discussing earlier about, your personal life, your friends and family, and you might adapt to how they are. So if a parent is eating fast food almost every day, then the child might say, “Actually, do you know what? That’s OK because my dad is doing it.’*

(NE2.2)

However, again this needs to be understood alongside young people’s foregrounding of the influence of economic and environmental factors on health practices, particularly in relation to food. 

#### 3.2.3. Interrelationships between Factors Linking Socioeconomic Position and Health

Young people’s discussions consistently highlighted the interrelationships between factors linking socioeconomic circumstance and health. The complex aetiology of health inequalities was both directly and indirectly acknowledged with understandings rooted in experience. Highlighting their focus on the interrelationship between different factors and the pathways through which inequalities were created and perpetuated, they articulated pathways between root causes (such as local labour market precarity) and secondary factors (such as not being able to afford to eat healthily):

*‘I think obviously because there’s high rates of unemployment and that links to not having money and then not like spending loads, that all links into like buying the cheapest food, which is not naturally healthy. So it all kind of links really.’*

(SY1.1)

The phrase ‘it all kind of links really’ encapsulates young people’s emphasis on the interwoven nature of inequalities. However, they consistently foregrounded poverty as the root cause of socioeconomic patterned inequalities in health: *‘if you don’t have a very good income then you can’t really live in a very good house. It can affect your health as it is and can cause like, it can cause stress which can cause other things*’ (SY1.2). The bounding and constraining impacts of stretched financial resources upon health practices and outcomes were clearly highlighted: ‘*I feel like money is one of the biggest factors for nearly everything, diet, mental health*’ (SY1.3). However, while in general young people’s narratives demonstrated their awareness of the socioeconomically disadvantaged nature of their local area, at times their discussions hinted that they associated poverty with others rather than themselves. In particular, one participant from one of the North East groups, the most socioeconomically disadvantaged area we worked in, noted: ‘*The less fortunate could actually find it harder … they’re not as privileged as we are in terms of money and wealth*.’ (NE2.3).

Whether they explicitly made the link or not themselves, young people’s narratives illuminated the importance they attached to the impact of poverty on mental health, typically the everyday, chronic stress and strain of living in poverty. They highlighted particular pinch points where limited financial resources were acutely stressful: ‘*I think there’s a certain level of stress if you go knowing that you’ve maybe not got as much money and there’s going to be certain times of the month where you have to really mind what you’re spending*’ (NE1.2). Mental health was perceived to be a consistent ‘link’ within a causal cycle of inequality—linked both to a decreased likelihood of engaging in healthful behaviours (such as eating well, engaging in exercise and labour market engagement) and an increased likelihood of engaging in risky behaviours (such as drinking and taking drugs):

*‘mental health is, like, connected to so many other things…like, reducing physical activities, and diet, and stuff like that. So mental health is, kind of, like, it could be, like, a major cause for the other things to happen so, like, comfort foods, for example, eating when you’re, like, depressed or something, or not getting out of bed due to, like, lack of food, like, due to depression so physical activity is just lower.’*

(L2.3)

## 4. Discussion

### 4.1. Social Determinants Shaping Health: Interacting Factors and Complex Pathways 

Through the course of their discussions, participants in our study often demonstrated nuanced understandings of how socioeconomic circumstances shaped health outcomes. Young people’s narratives showed how they were making sense of inequalities in health as they talked—at times echoing but, crucially, moving away from more populist individualised, neoliberal explanations for inequalities. Understandings of the relationship between socioeconomic circumstance and health, then, were not fixed and static but rather malleable and dynamic [17]. Overall, their narratives demonstrated a subtle appreciation of the ways in which the SDH get ‘under the skin to shape health’ through ‘interacting material, psychological and behavioural pathways’ (Raphael’s (2011) Discourse Level Three) [12] (p. 226). 

#### 4.1.1. Material Pathways 

Young people demonstrated an acute awareness of how differential access to material resources shaped opportunities to eat healthily, access health-promoting spaces and activities and enjoy good housing. Poverty was perceived to be all-pervasive, and young people consistently emphasised limited financial resources as a major barrier to health [48]. However, they also emphasised how this was exacerbated by other factors such as local infrastructures and perceived safety [49]. They also highlighted the uneven socioeconomic patterning of time—a finding not foregrounded by previous research exploring public perspectives of socioeconomic circumstances and health [17]. Their descriptions of the everyday stresses for low-income parents managing unsociable hours and caring responsibilities, particularly in relation to providing healthy food, resonate strongly with Strazdin et al.’s (2016) call to consider time as a social determinant of health as it has the potential to affect so many opportunities for good health—including time to engage in health-promoting activities, rest and care for each other [50]. The participants’ emphasis on time perhaps also hints at a weakness in the SDH framework which deals with social ‘domains’ and determining factors, rather than the mechanisms through which inequalities are sustained. 

#### 4.1.2. Psychosocial Pathways 

Young people consistently highlighted the importance of psychosocial mechanisms linking socioeconomic circumstance and health inequalities. They discussed the importance of mental health as a critical element in understanding the pervasive, complex influence of socioeconomic circumstance on health behaviours, experiences and outcomes (Discourse Level Three) [12]. Echoing previous studies with mostly adult participants this was particularly poignant in relation to housing [39,51,52,53,54]. Young people described both acute and chronic stress of living in inadequate housing, including the associated shame and stigma [39], offering poor housing as an important reason for higher rates of mental ill health among lower socioeconomic groups (Discourse Level Three) [12]. Participants’ discussions regarding socioeconomic inequalities in access to safe, green spaces and the ‘complex mix of spatial and social intertwinings’ also highlighted the impact on mental wellbeing [55] (p. 8). Such understandings contrast with findings from the recent Frameworks Institute project where participants foregrounded a ‘mentalism’ model in which ‘mental health issues such as depression and anxiety […] were seen as being determined by an individual’s mindset’ (their self-discipline and willpower) [18] (p. 7). 

#### 4.1.3. Behavioural Pathways 

At times, and particularly early on in discussions, young people emphasised the uneven patterning of risky health behaviours between socioeconomic groups, particularly in relation to substance use, smoking and alcohol (Discourse Level Two) [12]. This resonates with survey-based data with adults [18,53,56], and some qualitative work with both adults and young people [18,26]. Importantly, however, in the context of their discussions, young people’s narratives in our study frequently shifted towards a subtler appreciation of the role of the SDH, emphasising material and environmental factors as underpinning health behaviours. This finding differs markedly from recent UK-based research which characterises public understandings as focusing on personal responsibility and contrasts this with expert opinion (among those working in the field of social determinants) that behaviours are the very ‘endpoint in a long chain of causes and consequences that produce health outcomes’ [18] (p. 7). The young people’s accounts in our study resonate much more closely with the ‘expert’ understandings. They also echo the ‘integrated explanations for socioeconomically patterned inequalities’ evident among the mainly adult participants in Smith and Anderson’s (2018) meta-ethnography [17]. Importantly, however, both the participants in our study and the majority of those in the studies in Smith and Anderson’s (2018) review lived in socioeconomically disadvantaged areas and thus had personal experience of how inequalities played out in everyday life [17]. This may well explain the divergence. 

### 4.2. The Role of Public Policy Decisions (and Their Underlying Ideologies) 

While young people did not explicitly discuss the intersection between material living circumstances and gender or race (and only rarely referred directly to ‘class’ (Discourse Level Four) [12]), young people’s narratives sometimes demonstrated a critical consciousness of the role of public policy decisions and their underlying political philosophies in creating and sustaining inequity (Discourse Levels Five and Six) [12]. However, this was only really evident in their discussions regarding uneven geographic labour market precarity and the absence of regeneration investment [57]. A lack of political will to invest in the North (and vested interests in ensuring the success of the South) and underinvestment in certain local areas were directly blamed for reducing opportunities for good work and living conditions and, ultimately, good health [58,59]. The narratives echo previous research in which adult participants perceived some policies to be more favourable to some groups than others [14,51,60]. Our participants were also acutely aware of the unequal impact of the economic fallout of the COVID-19 pandemic on already disadvantaged young people [61], echoing research showing that young people suffer disproportionate impacts upon their employment trajectories and wages when exposed to economic uncertainty [62]. Young people’s emphasis on the unacceptability of poverty and scale of inequality contrasts with earlier studies (e.g., Shildrick and MacDonald’s 2013 study [47]). But reflects broader shifts in societal attitudes with ‘both phenomena being more widely regarded as prevalent and unacceptable than in the past’ [63] (p. 164). However, in general, there was much less evidence that participants spoke to Raphael’s Discourse Level Seven—about the power imbalances that underpin the uneven distribution of the SDH [12]. Health inequalities were described in relation to slightly abstract or faceless phenomena such as unemployment, poverty and regional inequality, but there was very little discussion about who has the power and how it is used to privilege some and marginalise others.

#### Determinants of Health Inequalities? 

While young people’s narratives offered apparently little space to disrupt the pathways between socioeconomic insecurity and health inequality, somewhat paradoxically, young people at times positioned themselves as avoiding the inevitability of this. Area fatalism and individual agency to resist risky health behaviours, for example, sat side by side. This was particularly evident in relation to (avoiding) substance use. Their emphasis on ‘room for agency’ to some extent echoes concerns about the language of social ‘determinants’. McMahon (2021) highlights that such a framing can perpetuate a reductionist approach to health inequalities [64]. Taken to its logical endpoint, this reduces individual people to ‘puppets on a string’ [65] (p. 475) and loses sight of the interaction between individuals, services, materiality and health [22]. The tension, however, was much less evident in relation to eating healthily and engaging in health-promoting activities where young people were more likely to share personal stories of the barriers they themselves faced [66]. This perhaps links to a greater acknowledgment of the bounding influence on poverty in relation to the food and exercise within public discourses more broadly. Indeed, at the time of the focus groups, a campaign for free school meals, led by Marcus Rashford, a prominent English football player, was the centre of much media attention [67], and the unequal impact of COVID-19 on people’s everyday living and working situations was very much in the spotlight [68]. 

Further, our analysis of young people’s emphasis on the interrelationships between pathways to inequalities also supports calls to move away from depicting discrete categories of determinants in relation to health inequalities [69]. Indeed, Dahlgren and Whitehead (2021) highlight that their rainbow model was only ever meant to depict determinants of health, not determinants of health *inequalities* [70]. To fully understand the root causes of health inequalities, they argue, we need to ‘take a further conceptual leap and focus on the pathways and mechanisms by which […] determinants […] bring about social gradients in health’ [70] (p. 22). Focusing on pathways and mechanisms in this way may also help to address the thorny issue of adequately articulating how health-relevant practices are constrained by people’s social and economic environment without inadvertently disempowering and further stigmatising underserved communities [64].

### 4.3. Study Limitations and Strengths 

Our sample of young people from socioeconomically deprived areas may limit the relevance of our findings for young people from more affluent areas. It also plays into a wider critique that by focusing on areas of socioeconomic deprivation such areas are perceived as the only communities in which inequality matters [17]. Further, while our sample as a whole is ethnically diverse, all participants in our North East and South Yorkshire groups were White British. 

Our decision to prioritise participant confidentiality also means that we have not provided individual participant demographic information alongside quotes. While this limits our ability to explore the extent to which individual participants held different views and the ways in which their understandings may have developed in the course of the discussions, we believe our commitment to confidentiality helped to facilitate young people’s engagement and openness during data generation. We were guided by a desire to ensure young people felt able to talk as freely as possible in the focus group setting. Indeed, we appreciated the limits of confidentiality in group discussions and therefore framed our questions in ways that ensured participants did not have to disclose personal information if they did not wish to and encouraged them to talk generally about people in their areas in light of this. This often resulted in discussions about their experiences and perspectives framed around ‘(some) young people’. 

It is also important to acknowledge the potential limitations of recruitment through existing youth organisations. Many youth organisations undertake work around health; therefore participants may have had more awareness about health inequalities than other groups of young people. Nevertheless, working closely with youth groups afforded many benefits. Youth workers helped to refine our topic guides and facilitate participant engagement, and they provided an invaluable source of trusted support for participants (see Woodrow et al., 2021 [31]).

Our approach of using three interlinked focus groups provided an opportunity to develop rapport, sense check and build on ideas over the sessions. The supportive atmosphere of the focus group in which young people were surrounded by peers and youth workers they knew, as well as research team members experienced in working with young people, perhaps helped to foster a more critical take and to enable participants to challenge each other. The context afforded young people a forum in which to develop understandings rather than being solely a means of extracting ideas. This highlights the importance of giving young people time and space to discuss and reflect on their perspectives on health inequalities [71,72]. Perhaps most importantly, we received consistently positive feedback from both participants and youth leaders across the three areas. Indeed, the retention of our participants over the series of three focus groups, which involved young people actively joining to participate in their free time (both whilst at home and during their youth groups sessions), demonstrates their engagement with and commitment to the project. 

Generating data during the COVID-19 pandemic also afforded a unique lens through which the young people viewed and subsequently discussed inequalities in health. Indeed, many young people recognised the unequal impact of the pandemic on health and were, to some extent, aware of the way existing inequalities have been exposed by the pandemic [11]. Therefore, this may help explain some of our findings around young people’s nuanced appreciations of the links between socioeconomic position and health.

### 4.4. Priorities for Future Research 

More research exploring young people’s perspectives on the relationships between socioeconomic circumstances, inequality and health is needed to address the current paucity. In particular, work with marginalised groups (such as looked-after children, care leavers, homeless young people, young people not in education, employment or training) who may be more likely to experience adverse social determinants of health would be beneficial [73]. Conversely, work with young people from more affluent contexts would provide interesting comparison and help counter a more general focus in the literature on areas of socioeconomic deprivation [17]. Further, research with groups not recruited through youth organisations would help explore if the perspectives found in our work were shaped by the participants’ involvement in youth organisations. Finally, it would be beneficial to explore ways to more effectively discuss, describe and teach topics of health inequality and look at ways to explore such topics in ways that are not stigmatising or fatalistic but that encourage positive social change [71]. 

### 4.5. Policy and Practice Implications

Our study highlights an ongoing need for policies that address young people’s everyday socioeconomic realities and experiences. First and foremost, young people’s emphasis on the all-pervasive impact of poverty on their opportunities to enjoy good health underscores the importance of pro-equity policies to end poverty. Their foregrounding of the uneven socioeconomic patterning of time and its impact on health and wellbeing highlights a need to tackle long (and often unsociable) working hours for people living in the most deprived neighbourhoods [74]. Further, there is an ongoing need for policies that address the conditions and impacts of unsuitable housing and that make it easier for young people, particularly those in socioeconomically disadvantaged areas, to eat more healthily and access health-promoting activities and spaces. 

While local authorities have responsibility to implement important practical changes here (e.g., enhancing green spaces and parks, making streets safer and establishing cycle lanes), this needs to be enabled by funding. The public health grant awarded to local authorities is currently one billion pounds lower (in real terms per capita) than it was in 2015/16 [24], and reductions in funding allocations have been higher in the poorest areas of the country [75]. In particular, young people in this study highlighted that youth clubs afford a safe space to socialise with peers, access information and advice and form trusting relationships with professionals. Yet, policy decisions have resulted in significant drops in funding for youth services with, for example, 750 youth centres forced to close between 2010/11 and 2018/19 [76]. This worrying trend has been exacerbated by increased funding pressures during COVID-19 [77]. Further, while on the one hand our study points to the importance of cross-sectoral action across a range of policy areas [46,78], we are wary here of falling into the trap of ‘shifting from a social inequality to a health inequality frame’ [79] (p. 653), and focusing our attention on the lower rather than the higher levels of Raphael’s (2011) seven discourses [12]. Such a framing, Lynch (2017) argues, can serve to make tackling inequalities seem like an insurmountable problem and divert attention away from policies (such as taxation, redistribution and labour market regulation) that we know will impact upon socioeconomic inequalities and, in doing so, health inequalities [79,80].

## 5. Conclusions

Our study affords an important contribution to the dearth of exploration around young people’s perspectives on inequalities in health [17,27,28]. Our focus on areas of high deprivation provides important insights and contributes to the limited body of work exploring the perspectives of people living on a low income in socio-epidemiological research more broadly [22,81] and calls for policy to tackle inequalities to be ‘grounded in the realities of people living in poverty’ [82] (para.2). Our study demonstrates that young people understand that health inequalities are generated by social determinants of health, which in turn influence behaviours. They highlight a complex interweaving of pathways between social determinants and health outcomes. However, they do not tend to think in terms of the SDH and their distribution as resulting from the power and influence of those who create and benefit from health and social inequalities. It may be that they are unused to thinking in this way or that they have understandings that we have not fully appreciated. An informed understanding of the causes of health inequalities, influenced by their own unique generational experiences, is important to help young people achieve greater equity in the future than they perceive at the present. 

## Figures and Tables

**Table 1:** Summary of Raphael’s (2011) seven discourses of the social determinants of health (SDH).

Discourse Level	Key Point
One: SDH as identifying and supporting those in need of health and social services.	Identifying and targeting those at greatest need through service provision.
Two: SDH as identifying those with modifiable medical and behavioural risk factors.	Identifying behavioural risk factors (e.g., diet, physical activity, alcohol and tobacco use) and promoting positive ‘lifestyle choices’.
Three: SDH as indicating the material living conditions that affect health.	Living conditions/circumstances affect health and choices either directly or indirectly through interrelated material, psychological and behavioural effects.
Four: SDH as indicating material living circumstances that differ as a function of group membership (class, gender and race).	Different (potential) axes of inequality can interact/intersect and compound each other to change people’s experience of the SDH.
Five: SDH and their distribution result from public policy decisions made by governments and other societal institutions.	Public policy can create and maintain (or reduce and disrupt) the SDH.
Six: SDH and their distribution result from economic and political structures and justifying ideologies.	Political and economic structures shape policy decisions.
Seven: SDH and their distribution result from the power and influence of those who create and benefit from health and social inequalities.	Individuals and groups shape policy that protects and benefits them at the expense of others (e.g., tax structures that favour the wealthy).

**Table 2:** Sample demographics.

Sample	Number of Participants	Age	Gender	Ethnicity	Deprivation Position
**Overall**	42	Age range: 13–21 Average age: 16.7	18 Female 19 Male 2 Non-binary 2 Trans Male 1 Gender-Fluid	30 White British 6 Asian/Asian British 3 Black/Black British 2 Mixed/Multiple ethnic group 1 Chinese	Average participant position = 8096 (Quintile 2)
**South Yorkshire 1** **(SY1)** **(urban)**	6	Age range: 15–17 Average age: 15.5	3 Female 2 Male 1 Gender-Fluid	6 White British	Average participant position = 8009 (Quintile 2)
**South Yorkshire 2** **(SY2)** **(urban)**	8	Age range: 13–17 Average age: 15.1	3 Female 5 Male	8 White British	Average participant position = 9414 (Quintile 2)
**North East 1** **(NE1)** **(rural, coastal)**	7	Age range: 15–17 Average age: 15.8	2 Female 1 Male 2 Non-binary 2 Trans Male	7 White British	Average participant position = 15004 (Quintile 3)
**North East 2** **(NE2)** **(rural, coastal)**	8	Age range: 13–20 Average age: 15.75	8 Male	8 White British	Average participant position = 1351 (Quintile 1)
**London 1** **(L1)** **(urban)**	10	Age range: 16–21 Average age: 18.7	8 Female 2 Male	1 White British 5 Asian/Asian British 3 Black/Black British 1 Mixed/Multiple ethnic group	Average participant position = 7065 (Quintile 2)
**London 2** **(L2)** **(urban)**	3	Age range: all aged 20 Average age: 20	2 Female 1 Male	1 Asian/Asian British 1 Mixed/Multiple ethnic group 1 Chinese	Average participant position = 7734 (Quintile 2)

## Data Availability

Data available on request due to restrictions. The data presented in this study are available on reasonable request from the corresponding author. The data are not publicly available due to privacy reasons.

## Supplementary Materials

The following supporting information can be downloaded at: https://www.mdpi.com/article/10.3390/ijerph19063679/s1, Supplementary File S1: Topic guides; Supplementary File S2: Participatory map; Supplementary File S3: Coding framework.
