# Supplementary material for: ‘It All Kind of Links Really’: Young People’s Perspectives on the Relationship between Socioeconomic Circumstances and Health
Source: Int J Environ Res Public Health. 2022 Mar 19;19(6):3679. doi: 10.3390/ijerph19063679 (PMC8950291; doi:10.3390/ijerph19063679)
Supplement: Supplementary file 1 [file ijerph-19-03679-s001.zip › Supplementary File 1 Topics Guides.pdf]

Focus group Topic Guides

The workshops will be delivered face-to-face if possible (dependent upon the Youth Group policies/practices and social distancing measure at the time of data collection), but are designed to run as online workshops if required.

Group information

- 3x 90 minutes focus groups with the same group of young people aged ≈ 13-17

- 8-10 participants, with breakout groups of 4-5
- Online platform [Google Meet / platform used by youth groups].
    - Online platform for interactive activities (e.g., Jamboards) enables online whiteboard to collect written data if online. Will be used via screen share function.
    - Participants can use chat/text feature if preferred (a moderator will be responsible for looking after the chat box)

Project overview

This project aims to enable children and young people (CYP) to be involved in action to reduce inequalities in the social determinants of health.

We will explore CYP views of health, including what makes people healthy and what makes it harder to be healthy and their perspectives regarding inequalities in health within their local area. We will work with CYP to identify their key priorities in addressing the impact of social determinants on their current and future health, and the messages that they would like to convey to people with a role in addressing health inequalities at a ‘place’ level. We will explore their perspectives through a combination of participatory methods, including mapping exercises. We will seek to work with children and young people who are themselves experiencing disadvantage and seeking inclusion.

Mobilising this understanding of CYP perspectives, we will work with a group of CYP and other key collaborators (including Public Health England, the Association for Young People’s Health and the Health Foundation) to co-produce a toolkit of multimedia resources aimed at equipping children and young people with an understanding of health and wellbeing as a human right, an understanding of the social determinants of health and ways to enable their active involvement in action to reduce inequalities in the social determinants of health.

Focus group 1

Focus group 1 (objective 1): Explore children and young people's <u>views of health</u> and <u>what makes people healthy and makes it harder for some people to be healthy within local places</u>

<table>
<tr><td></td><td>Focus group 1: Health in local places</td></tr>
<tr><td>Introduction in main room
(10 minutes)</td><td>• welcome/introductions/roles
• Study aims
• Key points from info sheet
• Ground rules for focus group discussion
    o Respect others views / one person speaking at a time
    o Confidentiality
    o Breaks if needed / can stop at any point without reason
        ▪ Any others to add?
• Discuss workshop plan
    • Systems mapping exercise
• Questions?
    • [if online - check everyone understand online platform functionality + check connection issues] - [if online - can use chat box]</td></tr>
<tr><td>Warm-up Activity
(20 minutes)</td><td><u>Favourite places in local area</u>

• [Moderator asks each young person]
• Introduce yourself, tell us your name and one of your:
    o favourite places in your local area that supports your health/wellbeing
    o Favourite places where you hang out/like to spend your time

    o Where do you spend your time
    o What do you do
        ▪ (Home / School / Sport / Online / Food places / With friends)

    • How do these things (if at all) relate to or impact your/others health

• [Moderator to summarise on if/how mentioned places are linked/joined up, and ask young people to expand on this]</td></tr>
<tr><td colspan="2">Break (10 minutes)
[Split into smaller groups / breakout groups - all facilitated]</td></tr>
</table>

<table>
<tr>
<td>Activity:
Systems mapping exercise</td>
<td><u>Introduction of Systems Mapping exercise</u>

We’d like to work together to create a ‘mind map’ of all the things that you think impact young people's health, and how they link together.

First, we want you to note down all the things you think are important for young people’s health in your local area.

There’s no bad ideas, write down anything you feel is important
These might be things which help/make it harder to be healthy

We’ll take it in turns to share our ideas and then we’ll think about how these different things might link together and be related (so does one impact another, and how).</td>
</tr>
<tr>
<td>Discussion in break out room
(30 minutes)</td>
<td><u>What influences young people’s health in your local area</u>

• [Moderator asks each young person]
• Please list all the things you think are important to/makes a difference to/impacts upon young people's health in the <u>local area</u>?
    o [each to spend a few minutes making a list of things which influence health in their local areas - take it in turns to share an idea + discuss if/how it links to other ideas]

    o What kind of things where you live support young people to be ‘healthy’ / make it harder to be healthy?
        ▪ Healthy choices/options/activities in [area
        ▪ Services (health, education, transport etc
        ▪ Local environment (green space, air quality
        ▪ Behaviours (activity, diet)
        ▪ Influences (peers, family, community etc.

    • Main focus / prompt
        ▪ So, what makes it easier/harder to be healthy in your local area?
            ▪ Why is this?
            ▪ What is it about X which is impacting health
        • How do these things link together - how does one impact the other

• Ask for a volunteer to feedback key points from discussion when return to whole group</td>
</tr>
<tr>
<td>Close in main room
(20 minutes)</td>
<td>[Bring groups back together for close]</td>
</tr>
</table>

- Volunteer participants to summarise some key points from the discussion, prompted/supported by facilitators as required (share Jamboard using screen share)
- [Moderators to record and summarise main points for discussion]
    - Any comments?

    - The participants will be provided with an opportunity to ask any questions, add anything we have missed or raise any points (both to the groups, and directly to research team)
    - The participants will be thanked for their participation, and it will be acknowledge that this is a difficult topic to speak about
    - Debrief + contact information for researchers / supporting organisations / youth worker for after care

- If face-to-face, Complete mini-demographic survey (return to researcher/youth worker)

[cool down activity]

- [Moderator to ask everyone to pick an emoji that best describes how they're feeling after the session]

At 90 minutes the session is scheduled to end.

- Thanks
    - Information + details about next workshop (time, access)
        - Encouraged to bring any relevant ideas/pictures/etc. To next meeting

Focus Group 2

Focus group 2 (objective 2): Explore children and young people's <u>understandings of inequalities in health</u>

<table>
<tr><th></th><th>Focus group 2: Understandings of inequalities in health</th></tr>
<tr><td>Introduction
(10 minutes)</td><td>

welcome/introductions/roles
Study aims
Key points from info sheet
Revisit ground rules for focus group discussion

Respect others views / one person speaking at a time
Confidentiality
Breaks if needed / can stop at any point without reason
Any others to add

Discuss workshop plan
Newspaper article discussion
Questions?

[if online - check everyone understand online platform functionality + check connection issues]
[if online - can use chat box]

Introduction of young people - all to say their names and something about themselves

</td></tr>
<tr><td>Warm-up Activity
(20 minutes)</td><td>
<u>Where do you stand?</u>

[Moderator to present health related statements to the young people, they have to vote if they think they agree/disagree/are not sure, then discuss]

Thumbs up if you agree with the statement, thumbs down if you disagree, wave an open palm if you are not sure.

E.g.,
Young people are the main spreaders of COVID-19
McDonalds is better than KFC
Being healthy is easy where I live
There is enough support for young people mental/physical health in school/in the community
People already have everything they need to be healthy

</td></tr>
</table>

<table>
<tr><td></td><td>Why do you think this?</td></tr>
<tr><td colspan="2">Break (10 minutes)</td></tr>
<tr><td colspan="2">Split into smaller groups / breakout groups - all facilitated</td></tr>
<tr><td>Activity:
Health Inequality discussion & Presentation of health inequalities media/news articles/infographics prompts</td><td><u>Understandings of inequalities in health</u>

We want to look at your understanding of inequalities in health. To do this we will explore what you think health inequalities are, and then we are going to present some news articles about health inequalities, and would like you to select some, and discuss what you think about them. Then we’d like you to suggest your own alternative headlines which you think are more relevant.</td></tr>
<tr><td>Discussion: (30 minutes)</td><td>• What does ‘inequality’ mean to you?
• What kind of inequalities in health do you think there are/could be/do you know about
    • What things do you think can influence these

• [The moderator will present (screen share) and read NHS definition of health inequality as prompt if needed.
• NHS definition: ‘Health inequalities are unfair and avoidable differences in health across the population, and between different groups within society. Health inequalities arise because of the conditions in which we are born, grow, live, work and age. These conditions influence our opportunities for good health, and how we think, feel and act, and this shapes our mental health, physical health and wellbeing’

• [Moderator to present selection of news articles / infographics / social media articles around health/health inequalities - let young people select ones to speak about, and explain why these have been chosen]

    • What are these articles saying
        o Is there anything unfair about the headlines? Why?

    • Why do you think these health inequalities may exist/persist</td></tr>
</table>

	• Do you feel the headlines apply to your life and friends?<br><br>• Main focus / prompt<br>• **What kind of health inequalities do you think there could be where you live? - Why?**<br>• **What things where you live help stop / contribute to such issues/health inequalities?**<br><br>• ~~Correct the headlines~~<br>o ~~Make the headlines more fair and rewrite one/some of them to be more representative of your experience of being a young person~~
Close<br>(20 minutes)	[Bring groups back together for close]<br><br>• Volunteer participants to summarise some key points from the discussion, prompted/supported by facilitators as required<br>• [Moderators to record and summarise main points for discussion]<br>o Any comments?<br><br>o Discuss how health inequalities are evident across all aspects of society, and are not deterministic<br>o Things are being done to correct them - we will discuss these is the next session<br><br>o The participants will be provided with an opportunity to ask any questions, add anything we have missed or raise any points (both to the groups, and directly to research team)<br>o The participants will be thanked for their participation, and it will be acknowledge that this is a difficult topic to speak about<br>o Debrief + contact information for researchers / supporting organisations / youth worker for after care

	[cool down activity]<br><br>• [Moderator to ask everyone to pick an emoji that best describes how they're feeling after the session]<br><br>At 90 minutes the session is scheduled to end.<br><br>• Thanks<br>○ Information + details about next workshop (time, access)<br>▪ Encouraged to bring any relevant ideas/pictures/etc. To next meeting

Focus Group 3

Focus group 3 (objective 3): Work with CYP to identify <u>key priorities for CYP</u> in addressing the impact of social determinants on their current and future health, and the <u>messages that they would like to convey</u> to people with a role in addressing health inequalities at a 'place' level

<table>
<tr><td></td><td>Focus group 3: Key priorities and Change</td></tr>
<tr><td>Introduction
(10 minutes)</td><td>• welcome/introductions/roles
• Study aims
• Key points from info sheet
• Revisit ground rules for focus group discussion
    o Respect others views / one person speaking at a time
    o Confidentiality
    o Breaks if needed / can stop at any point without reason
        ▪ Any others to add
• Discuss workshop plan
    • Policy Discussion
• Questions?
    • [if online - check everyone understand online platform functionality + check connection issues]
    • [if online - can use chat box]</td></tr>
<tr><td>Warm-up Activity:
(20 minutes)</td><td><u>Top tips for good health</u>

• We would like your ‘top tip’ for good health and wellbeing - what would be the one most important thing you’d say as a piece of advice to be healthy
• Do you follow this?</td></tr>
<tr><td colspan="2">Break (10 minutes)</td></tr>
<tr><td colspan="2">Split into smaller groups / breakout groups - all facilitated</td></tr>
<tr><td>Activity:
Discussion of key priorities for young people regarding health and wellbeing where they live + systems map</td><td><u>Your key priorities for young people’s health where you live</u>

We want to explore what you think are the most important things for improving young peoples’ health where you live. We’re going to ask you what things you would like to see changed to improve young people’s health where you live, and why.</td></tr>
<tr><td>Discussion</td><td>• [Moderator asks each young person]</td></tr>
</table>

<table>
<tr>
<td>(30 minutes)</td>
<td>If you were the mayor/in charge of the local council, what would you do in order to improve the health of young people where you live ‘
• Why?
• How do you think this would work?

• What kinds of things are being done / already out there for young people in your local area
o What should be done
o Who should do it

• [Moderator to share systems map for discussion]
o This is the map we made from our discussions in the first session when we discussed being healthy in your local area.
o It helps us see how different factors are linked. What do you think are the important factors to change looking at this?

• Main focus / prompt
o What would be the most important things to change to improve young people's health where you live</td>
</tr>
<tr>
<td>Close
(20 minutes)</td>
<td>[Bring groups back together for close]

• [Moderators to record and summarise main points for discussion]
o Any comments?

o Discuss how health inequalities are evident across all aspects of society, and are not deterministic
o Things are being done to correct them - we will discuss these is the next session

o The participants will be provided with an opportunity to ask any questions, add anything we have missed or raise any points (both to the groups, and directly to research team)</td>
</tr>
</table>

	○ The participants will be thanked for their participation, and it will be acknowledge that this is a difficult topic to speak about<br>○ Debrief + contact information for researchers / supporting organisations / youth worker for after care<br><br>[cool down activity]<br><br>• [Moderator to ask everyone to pick an emoji that best describes how they're feeling after the session]<br><br>At 90 minutes the session is scheduled to end.<br><br>• Thanks<br><br>• Information about what happens next in the research<br>○ Do you want to be kept updated on the research<br>▪ Our research will be completed by March 2022. If you would like to hear what we found out you can visit https://sphr.nihr.ac.uk/.<br>• How can we best keep you informed?
