# Supplementary material for: ‘It All Kind of Links Really’: Young People’s Perspectives on the Relationship between Socioeconomic Circumstances and Health
Source: Int J Environ Res Public Health. 2022 Mar 19;19(6):3679. doi: 10.3390/ijerph19063679 (PMC8950291; doi:10.3390/ijerph19063679)
Supplement: Supplementary file 1 [file ijerph-19-03679-s001.zip › Supplementary File 3 Coding Framework.pdf]

Coding Framework

Parent code	Child code	Grandchild code
**Factors affecting health**	Personal / household resources	• Food security<br>• Housing<br>• Personal transport<br>• Income/wealth/ affordability<br>• Education level (self/family)<br>• Welfare benefits (inc FSM & food banks)<br>• Equipment / home learning resources (books, IT, sports kit etc)<br>• Time<br>• Useful social connections<br>• Health knowledge
	Services /facilities	• Housing service<br>• Public transport<br>• Health services<br>• Community and leisure centres<br>• School (inc. mental health & cooking classes)<br>• Youth clubs<br>• Facilities (availability & affordability)
	Physical environment	• Safety and crime<br>• Aesthetic/reputation of environment (i.e. Positive / negative/ dodgy / rough etc).<br>• Accessibility (disability access)<br>• Noise/air pollution<br>• Favourite places<br>• Green spaces<br>• Active travel<br>• Food environment (access to takeaways etc)
	Relationships / People	• Family (support/stability/conflict)<br>• Friends as support / socialising<br>• 'Trusted adults' (youth workers/teachers)<br>• Other people's attitudes & behaviour (bullying, prejudice, discrimination, hate crime)<br>• Peer pressure/influence<br>• Social media<br>• Community & cultural /family/peer health norms and practices
	Socioeconomic context of the place	• Historical context<br>• Jobs (quality - quantity)<br>• Income (in)equality<br>• Politics<br>• Educational opportunities<br>• Government investment (national/local)<br>• Community cohesion<br>• Potential for social mobility
	Individual behaviours	• Smoking<br>• Drugs & Alcohol<br>• Healthy eating/diet

		• Physical activity<br>• Personal effort<br>• Participation (volunteering/helping others)
	Psychosocial	• Stigma<br>• Stress<br>• Shame /prejudice<br>• Unsatisfying job<br>• Social comparison (perceptions of income)<br>• Sense of control<br>• Fatalism (feeling like no point)<br>• Self-esteem/ body image<br>• Sense of belonging<br>• Coping strategies
	Interrelationship between factors	• Structural factors influencing individual behaviours (e.g. income – stress – mental health etc.<br>• people versus place<br>• Interrelationship physical and mental health

**Understandings health inequalities**	**Defining (health) inequality**	• **Sources of information**<br>• **Making sense of info (potential for partial understandings)**
	Axes of inequality	• Local area examples<br>• National examples<br>• International examples<br>• Income<br>• North/South<br>• Ethnicity<br>• Age<br>• Sexuality<br>• Gender<br>• Religion<br>• Disability / health status<br>• Weight<br>• Intersecting disadvantage<br>• Class
	Pathways of inequality (i.e. how one thing leads to another etc)	• Initial conditions (highlighted as important for health)<br>• Secondary factors (linking initial conditions to health impacts)<br>• Intergenerational patterns of inequality<br>• Pathway disruptors<br>• Pathway amplifiers
	Opinions on inequalities	
	Positioning in relation to different socio-economic group	

**Tackling inequalities in health**	**Priorities for change / potential mechanisms**	• **Wellbeing provision in schools**<br>• **Accessibility (to safe and healthy places)**<br>• **Healthy eating (affordability/accessibility/options & choice)**<br>• **Promoting physical health**<br>• **Youth Pay/wage**<br>• **Allocating funding / resources**<br>• **Policy implementation (How policies are implemented / how well they're working e.g. potential stigma re. FSM & NCMP)**<br>• **Tackling anti-social behaviour & crime**
	Responsibility for tackling inequalities in health	• Government<br>• School<br>• Parents<br>• Young people (& their perceived ability to influence change)
