# Supplementary figures and images for: ‘It All Kind of Links Really’: Young People’s Perspectives on the Relationship between Socioeconomic Circumstances and Health
Source: Int J Environ Res Public Health. 2022 Mar 19;19(6):3679. doi: 10.3390/ijerph19063679 (PMC8950291; doi:10.3390/ijerph19063679)

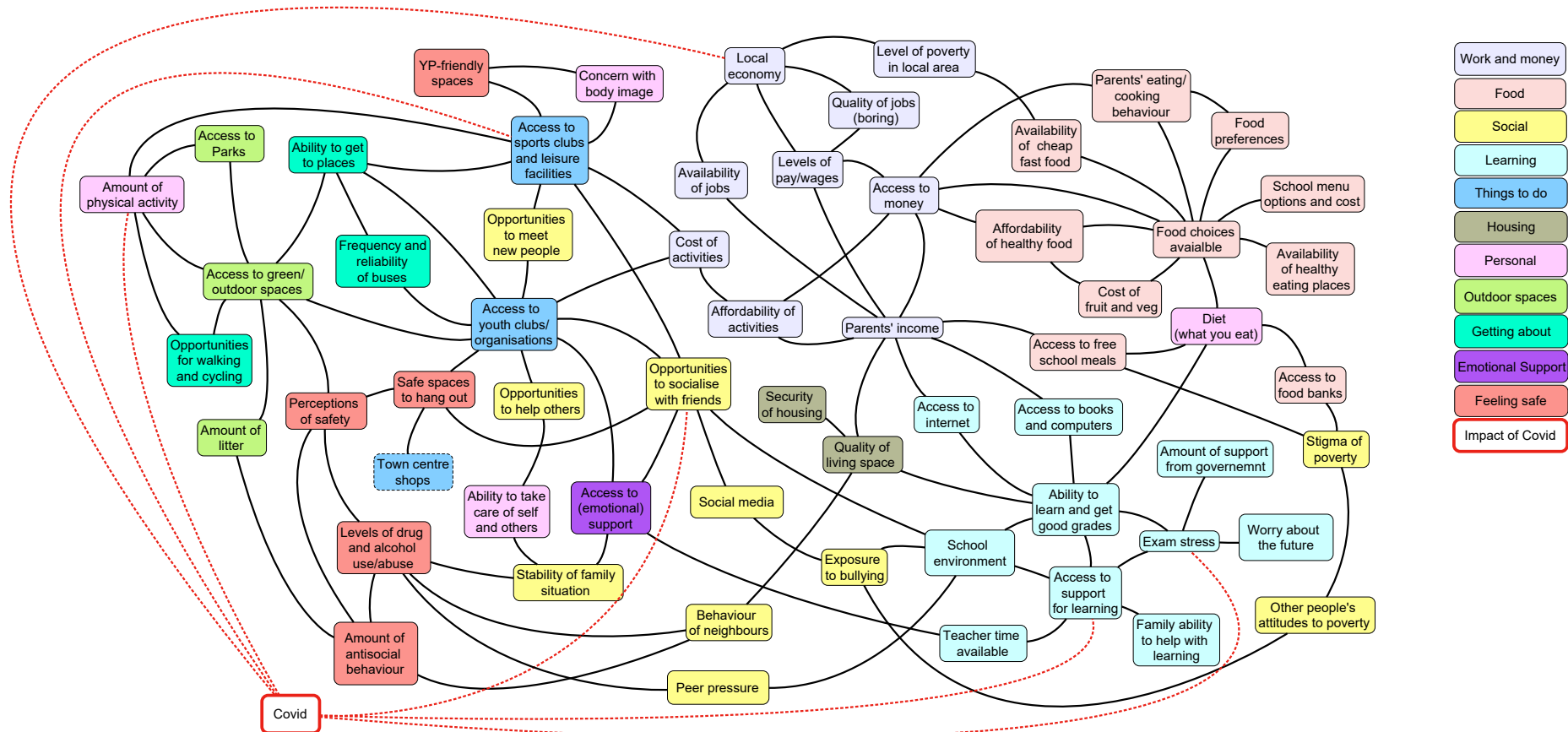

Supplement: Supplementary file 1 [file ijerph-19-03679-s001.zip › Supplementary File 2 Participatory Map.pdf]
